# Nephrotoxicity of Immune Checkpoint Inhibitors: Acute Kidney Injury and Beyond

**DOI:** 10.7759/cureus.12204

**Published:** 2020-12-21

**Authors:** Mohammad Tinawi, Bahar Bastani

**Affiliations:** 1 Nephrology, Nephrology Specialists Pc, Munster, USA; 2 Internal Medicine - Nephrology, Saint Louis University School of Medicine, Saint Louis, USA

**Keywords:** immune checkpoint inhibitors, onconephrology, cancer immunotherapy, acute kidney injury, acute tubulointerstitial nephritis, electrolyte disorders, fanconi syndrome

## Abstract

Immune checkpoint inhibitors (ICIs) are novel humanized monoclonal antibodies that release the brakes on the immune system, resulting in the destruction of tumor cells. ICIs are approved for a variety of hematological and solid organ malignancies, and the list has been growing since the approval of ipilimumab in 2011. ICIs are associated with a variety of immune-related adverse events (irAEs). irAEs commonly affect the skin, the gastrointestinal (GI) tract, and the endocrine system. Acute kidney injury (AKI) due to ICIs (ICI-AKI) occurs in a minority of patients, and it is usually due to acute tubulointerstitial nephritis (ATIN). Treatment with corticosteroids is usually successful. There have been reports of electrolyte disorders due to ICIs, including hyponatremia, hypocalcemia, hypokalemia, and Fanconi syndrome. The diagnosis of electrolyte disorders requires vigilance and routine laboratory monitoring.

## Introduction and background

Immune checkpoint inhibitors (ICIs) represent a major advance in cancer treatment over the past decade. Immunotherapy has revolutionized cancer treatment and is playing an ever-increasing role in the management of many malignancies. The list of approved indications for ICIs is steadily increasing. ICIs are new arrows in the quiver alongside surgery, chemotherapy, and radiation therapy.

T-cell activation requires two signals. The first signal is the interaction between T cell receptor (TCR) on T-lymphocytes and the major histocompatibility complex (MHC) on antigen-presenting cells (APC). The second signal is the interaction between B7 (CD 80/86) on APC and CD 28 on T-lymphocytes.

CTLA-4 (cytotoxic T-lymphocyte associated antigen 4) is a CD28 homolog on T-lymphocytes with a higher binding affinity for B7 (CD80/86) expressed on APC. CTLA-4 and B7 interaction inhibit T cell activation. CTLA-4 inhibitors, such as ipilimumab or tremelimumab, block this interaction and subsequently enhance T cell activation [1]. Programmed cell death protein-1 (PD-1) is a receptor on the surface of activated T cells that binds to PD ligand 1 (PDL-1) on the surface of tumor cells. This blocks TCR signaling and results in tumor cell proliferation. Inhibitors of PD-1 (pembrolizumab, nivolumab, cemiplimab) or PDL-1 (atezolizumab, avelumab, durvalumab) induce tumor cell apoptosis and T cell activation.

Ipilimumab, a monoclonal antibody that targets CTLA-4 and activates the immune system, was the first ICI approved by the U.S. Food and Drug Administration (FDA) in 2011 for the treatment of melanoma. Currently, there are seven ICIs on the market (Table 1).

**Table 1:** FDA-approved ICIs, their immune targets, and their common indications CTLA-4: cytotoxic T-lymphocyte associated antigen; PD-1: programmed cell death protein-1; PDL-1: programmed cell death protein ligand-1; FDA: Food and Drug Administration; ICIs: immune checkpoint inhibitors

ICI	Immune Target	Year Approved	Common Indications
Ipilimumab	CTLA-4	2011	Melanoma
Pembrolizumab	PD-1	2014	Non-small-cell lung cancer, melanoma
Nivolumab	PD-1	2014	Non-small-cell lung cancer, hepatocellular carcinoma, melanoma
Atezolizumab	PDL-1	2016	Non-small-cell lung cancer, urothelial carcinoma
Avelumab	PDL-1	2017	Merkel-cell carcinoma, urothelial carcinoma
Durvalumab	PDL-1	2017	Urothelial carcinoma
Cemiplimab	PD-1	2018	Metastatic cutaneous squamous cell carcinoma

As with any new therapy, it is critical to monitor for potential adverse reactions to these new agents. Renal function and electrolytes should be routinely monitored when administering ICIs because AKI and electrolyte disorders are encountered in some patients on ICIs immunotherapy, leading to significant complications.

This article explores different aspects of AKI and electrolyte disorders complicating ICI immunotherapy, including controversial issues such as performing kidney biopsy in patients with AKI due to ICIs.

The Common Terminology Criteria for Adverse Events (CTCAE) used for oncology drugs divides the AEs associated with these medications into five grades: grade 1 (mild), grade 2 (moderate), grade 3 (severe but not immediately life-threatening), grade 4 (life-threatening), and grade 5 (death related to AE).

In our discussion, we will use the AKI classification proposed by the Kidney Disease Improving Global Outcomes (KDIGO) (Table 2) [2].

**Table 2:** Stages of AKI according to KDIGO AKI: acute kidney injury; KDIGO: Kidney Disease Improving Global Outcomes

KDIGO AKI Stage	Serum creatinine	Urine output
Stage 1	serum creatinine 1.5-1.9 x baseline or an increase of ≥ 0.3 mg/dl within 48 h	< 0.5 ml/kg/h for 6-12 hours
Stage 2	serum creatinine 2-2.9 x baseline	< 0.5 ml/kg/h for ≥ 12 hours
Stage 3	increase in serum creatinine to ≥ 4 mg/dl or 3 x baseline or the initiation of renal replacement therapy (RRT)	< 0.3 ml/kg/h for ≥ 24 hours or anuria for ≥ 12 hours

## Review

Immune-related adverse events (irAEs) due to ICIs

The most common irAEs associated with the use of ICIs are related to the skin (rash, pruritus, Stevens-Johnson syndrome), the GI tract (gastritis, pancreatitis, ileitis, colitis), the endocrine system (hypothyroidism, hyperthyroidism, adrenal insufficiency, hypophysitis), and the respiratory system (pneumonitis, sarcoidosis-like granulomatosis, and pneumonitis) [3]. Renal involvement in the form of ICI-AKI is encountered less often.

The focus of this review article is the renal adverse effects of ICIs, including AKI and electrolyte disorders. We present data from several meta-analyses and clinical studies. We also included data from case series and case reports deemed of clinical significance. FDA adverse events database on electrolyte disorders are reviewed as well.

Incidence of ICI-AKI

Earlier reports of ICI-AKI performed kidney biopsy to establish the underlying pathologic diagnosis. Kidney biopsies were done because the clinical entity was new and its characteristics were not well-defined. Later, the American Society of Clinical Oncology published guidelines discouraging kidney biopsy and favoring empirical glucocorticoid treatment when ICP-AKI is suspected [4]. Later reports used the KDIGO AKI definition and did not require a kidney biopsy to ascertain the diagnosis in every case. Naturally, all cases of AKI had to be clinically adjudicated and other causes of AKI had to be excluded on clinical grounds. Some studies restricted the definition of ICI-AKI to stages 2 and 3 [5]. AKI stages 2 and 3 have more clinical significance with regards to subsequent clinical events. The incidence of stage 3 AKI, defined as an increase in serum creatinine > 3 times baseline or increase in serum creatinine to > 4.0 mg/dl, or the need for initiating RRT, was 0.6%. The authors did not report on RRT separately.

Manohar et al. conducted a meta-analysis involving 48 clinical trials that monitored renal function and serum electrolytes in a total of 11,482 patients while on treatment with pembrolizumab or nivolumab (both are PD-1 inhibitors) [6]. The overall risk ratio (RR) of ICI-AKI was 1.86 (95% confidence interval (CI) 0.95-3.64, P-value 0.07).

Cortazar et al. analyzed data from phase 2 and 3 clinical trials involving 3,695 patients [7]. The incidence of ICI-AKI was 3% and increased to 5% in patients on dual ICI therapy. The incidence of KDIGO stage 3 ICI-AKI was 0.6%.

Mechanism, risk factors, and pathology of ICI-AKI

The mechanism of ICI-AKI is unclear. There are speculations that ICIs can elicit an autoimmune response against a potential antigen on the surface of renal tubular cells resulting in ATIN [8].

The predominant lesion in patients who underwent a kidney biopsy to determine the etiology of ICI-AKI was ATIN [5,7,9-11]. This has been consistent in all reports to date. Other lesions have been rarely reported, such as immunoglobulin A (IgA) nephropathy, membranous nephropathy, and pauci-immune glomerulonephritis [11].

In the series reported by Cortazar et al., 138 patients had ICI-AKI, 43% of whom underwent a diagnostic kidney biopsy. In >90%, the pathology showed ATIN (20% of kidney biopsies had granulomatous ATIN) [4]. Three risk factors for ICI-AKI were identified: Concomitant use of proton pump inhibitors (PPIs), low baseline estimated glomerular filtration rate (eGFR) (< 30 ml/min/1.73 m^2^), and using dual ICI therapy. The authors did not report on the complications of renal biopsies. Interestingly, concomitant use of PPIs has been shown to be a risk factor for ICI-AKI in other reports too [7,9-12], and PPIs have been associated with an increased risk of ATIN in the general population [13].

There are no distinguishing features between ATIN due to ICIs and ATIN due to other causes. Patients with ATIN due to ICIs may present with microhematuria, subnephrotic range proteinuria, sterile pyuria, and rarely eosinophilia [5,9]. In all of the above-referenced studies, there was a significant latency between the initiation of therapy with ICIs and the development of ICI-AKI. Patients were treated with ICIs for 13-40 weeks before ICI-AKI was diagnosed. The latency of AKI is probably due to the prolonged longevity of activated T-cells, rather than the direct toxicity of ICIs [14].

A rare but potentially life-threatening adverse effect of ICIs is thrombotic microangiopathy. Few case reports have reported thrombotic thrombocytopenic purpura (TTP) in conjunction with ICIs immunotherapy. Recently Ali et al. reported a case of TTP in a patient treated with nivolumab and ipilimumab for metastatic renal cell carcinoma [15]. 

To biopsy or not to biopsy

The role of kidney biopsy in the diagnosis of ICI-AKI remains to be defined [16]. Some authorities recommend performing a kidney biopsy to ascertain the diagnosis in absence of a contraindication. Others recommend a kidney biopsy if a different etiology is suspected such as acute glomerulonephritis. Advocates of performing kidney biopsy remind us that kidney biopsy is the gold standard for establishing the diagnosis. If the cause of AKI is not ICI-related (for example, acute tubular injury (ATI)), the patient will avoid unnecessary treatment with corticosteroids and can be continued on ICIs without interruption of vital cancer immunotherapy [17]. Those who advise performing kidney biopsy only on a case-by-case basis argue that most ICI-AKI cases are due to ATIN and most patients are successfully treated with corticosteroids [18].

Management of ICI-AKI

ICIs should be stopped if KDIGO AKI stage 2 or 3 is diagnosed. Patients with KDIGO AKI stage 1 can be observed closely, and ICIs should be discontinued if AKI worsens. Corticosteroids are the treatment of choice for ICI-AKI, and they should be initiated in severe AKI cases as early as possible. If we combine the data from the above-referenced six studies [5,7,9-12] we can conclude the following:

The six studies included 215 patients with ICI-AKI, 82% (177 patients) were treated with corticosteroids, though many different regimens were reported. Some patients had received intravenous corticosteroids initially.

Of the patients who received corticosteroids, 79% had a partial or complete recovery. Some patients died of their primary malignancy while on corticosteroids.

A total of 52 patients were rechallenged with ICIs (some while still on corticosteroids), of whom 18 patients (35%) had a recurrence of ICI-AKI.

In the study by Cortazar et al., 31 patients were re-challenged, seven (23%) of whom developed recurrent ICI-AKI. Of the seven patients, only one did not recover from ICI-AKI [5]. At the time of re-challenging the patients, 12 of 31 patients were still receiving corticosteroids and 87% of patients were re-challenged with the same ICI. Since ICIs are essential in increasing survival in many cancer patients, it is reasonable to cautiously consider a rechallenge after the improvement of ICI-AKI.

When patients were treated with oral corticosteroids, a common practice was to start prednisone at 1 mg/kg and to taper it slowly over eight to 12 weeks.

Corticosteroids are the mainstay of treatment. Other immunosuppressive agents used were mycophenolate mofetil, rituximab, cyclophosphamide, and infliximab [5,11].

ICI use in kidney transplant and end-stage kidney disease (ESKD)

Venkatachalam et al. reported poor renal outcomes in kidney transplant patients treated with ICIs [19]. Their report included six kidney transplant recipients, with metastatic cancers unresponsive to first-line treatments. They received a variety of ICIs and three of the six patients developed AKI. Their AKI was due to biopsy-proven acute cellular rejection in one patient, mixed acute cellular and acute antibody-mediated rejection with areas of cortical necrosis in another patient. In the third patient, acute rejection was diagnosed on clinical grounds and kidney biopsy was not done; his renal function improved after discontinuation of ICI therapy. Cancer progressed in five of the six patients, and only one patient had remission.

There is a paucity of data on the use of ICIs in patients with ESKD. Hirsch et al. reported eight patients with ESKD (seven patients were on hemodialysis, and one patient was on peritoneal dialysis) who were treated with ICIs [20]. There were no dose adjustments and all eight patients were concomitantly receiving PPIs. Two of the eight patients developed ICI-related dermatitis and were treated with corticosteroids and discontinuation of ICIs. Both patients had cancer progression. The remaining six patients tolerated ICIs well. Eventually, cancer had progressed in five of the eight patients and four of them had died at the time of preparing the report.

The characteristics of ICI-AKI are summarized in Table 3.

**Table 3:** Characteristics of ICI-AKI ICI-AKI: immune checkpoint inhibitor-associated acute kidney injury; ATIN: acute tubulointerstitial nephritis; PPI: proton pump inhibitor; eGFR: estimated glomerular filtration rate; ESKD: end-stage kidney disease

Incidence	2%-5%
Mechanism	Unclear
Risk factors	PPIs use, dual ICI therapy, eGFR < 30 ml/min/1.73 m^2^
Dominant pathology	ATIN
Role of kidney biopsy	Controversial. A biopsy is indicated if an alternate diagnosis is suspected.
Management	Discontinuation of ICIs and initiation of corticosteroids for ATIN
Rechallenge post-ICI-AKI	Can be considered on a case by case basis
Use in kidney transplant recipients	Early data show poor outcome due to acute rejection
Use in ESKD	Early data are encouraging

Electrolyte disorders due to ICIs

Electrolyte disorders are important adverse reactions of ICIs. Examples include hyponatremia, hypocalcemia, hypercalcemia, hypomagnesemia, hypokalemia, hyperkalemia, hypophosphatemia, and Fanconi syndrome.

The meta-analysis by Manohar et al. included five randomized controlled trials (RCTs) that assessed the association between PD-1 inhibitors and electrolyte disorders [6]. In patients treated with PD-1 inhibitors, the overall pooled RR of electrolyte disorders was 1.67 (95% CI 0.89-3.12, P-value 0.11). Hypocalcemia was more prevalent. The pooled incidence of hypocalcemia with PD-1 inhibitors therapy was 1.0% (95% CI 0.6-1.8%). Hypocalcemia reached a severe degree (CTCAE grade 3 or higher) in 13% of cases (95% CI 3.3-39.4%). In the same meta-analysis, two studies with pembrolizumab were analyzed and showed hypocalcemia pooled RR of 10.9 (95% CI 1.40-84.16, P-value 0.02). The mechanism of hypocalcemia is unknown and there was no data on vitamin D or intact parathyroid hormone (PTH) levels. There was no significant association between PD-1 inhibitors and other electrolyte disorders, including hypercalcemia, hypomagnesemia, hypokalemia, and hyponatremia. Despite the absence of a significant association, Manohar et al. found that the reported electrolyte disorders were CTCAE grade 3 or higher in 28.8% of cases. Moreover, while they could not find a significant association with hyponatremia, 53% of electrolyte abnormalities reported as CTCAE grade 3 or higher were hyponatremia, and one case of hypercalcemia resulted in death (CTCAE grade 5).

Wanchoo et al. reviewed the FDA adverse events database from the third quarter of 2011 until the first quarter of 2015 [21]. They identified 256 cases of renal injury and 312 cases of electrolyte disorders associated with the use of ipilimumab, nivolumab, and pembrolizumab. Hyponatremia was the most common (61.5%) electrolyte disorder, followed by hypokalemia (23%), hyperkalemia (8%), hypophosphatemia (4.5%), and hypomagnesemia (3%), there were no reports of hypernatremia. The authors did not report on the degree of hyponatremia or the severity of other electrolyte abnormalities. Of note, 83% of cases were associated with ipilimumab, which was the first FDA-approved ICI, the other two ICIs in the review were approved three years later. The proposed mechanism of hyponatremia was hypophysitis and secondary adrenal insufficiency. The authors did not report on whether there was a concomitant AKI in these cases.

There have been several case reports of severe electrolyte abnormalities associated with the use of ICIs. Win et al. reported a case of primary hypoparathyroidism in a 73-year old man with metastatic melanoma who presented to the emergency department with severe symptomatic hypocalcemia (serum calcium level of 5 mg/dL, iCa 0.67 mM) [22]. He had been started on ipilimumab and nivolumab 1.5 months prior to presentation. His PTH level was undetectable. He was treated with calcium and vitamin D. Interestingly, he developed thyrotoxicosis shortly after his hospital admission followed by primary hypothyroidism requiring thyroid hormone replacement therapy. The authors emphasized that the polymorphism of immune checkpoint genes has been associated with several autoimmune conditions such as Graves disease and myasthenia gravis [23].

Balakrishna et al. reported the case of a 58-year-old man with a history of metastatic melanoma who presented with progressive quadriplegia after 10 months of treatment with nivolumab [24]. Initial chemistry panel showed: potassium 1.7 mEq/L, bicarbonate 9 mEq/L, serum creatinine 2.6 mg/dL (baseline: 0.9 mg/dL three weeks prior), and 24-hour urine potassium 159 mEq/L. Arterial blood gases, magnesium, and phosphorus were not reported. The patient also had 6% eosinophilia. A renal biopsy was not done. The patient was treated with a four-week course of corticosteroids and nivolumab was discontinued. Potassium and bicarbonate were replaced. Quadriplegia resolved by the third hospital day and he was discharged with normal serum potassium, bicarbonate, and creatinine. He was then rechallenged with pembrolizumab, which he tolerated. It is conceivable that the patient had renal tubular acidosis (severe hypokalemia, low serum bicarbonate), but the report does not provide adequate data to ascertain that diagnosis.

The authors of this review have reported the first case of Fanconi syndrome associated with the use of ICIs (published online first in September 2019) [25]. The patient was a 59-year old man with hepatocellular carcinoma. He presented to the emergency department eight months after initiation of therapy with nivolumab, with a serum potassium of 1.7 mEq/L, bicarbonate 20 mEq/L, serum anion gap 10 mEq/L, and serum phosphorus 1.2 mg/dL. Serum concentrations of sodium, chloride, magnesium, calcium, and creatinine were essentially normal. Urine studies showed potassium 89 mEq/24 hours, glucose 100 mg/dL, and protein 1014 mg/24 hours. Fractional excretion of phosphorus was 11%. Urine quantitative amino acid analysis revealed a significant elevation of gamma-aminobutyrate and minimal to moderate elevations of several other amino acids. Urinalysis showed a urine pH of 7.0. Arterial blood gas: pH 7.39, partial pressure of carbon dioxide (PCO2) 25 mmHg, partial pressure of oxygen (PO2) 75 mmHg, bicarbonate (HCO3) 15 mEq/L, base excess -8 mEq/L, and O2 saturation 95% on room air. He received an aggressive intravenous and oral replacement of potassium and phosphorus, and nivolumab was discontinued. He was discharged after six days on oral K and phosphate supplements. His presentation was due to acquired Fanconi syndrome (proximal renal tubular acidosis with phosphaturia, glycosuria, and aminoaciduria) due to nivolumab. He was not treated with corticosteroids and a kidney biopsy was not done. The mechanism is unclear but probably related to ICIs' toxic effect on the proximal tubules.

Farid et al. published a second case report of Fanconi syndrome due to ICIs [26]. The patient was a 58-year old man with advanced stage small-cell lung cancer who presented with immune-related hepatitis three weeks after starting nivolumab and ipilimumab. The two ICIs were discontinued, and he was treated with corticosteroids and mycophenolate mofetil. Ten days later, he presented with abdominal pain and fever. His chemistry panel was significant for serum creatinine 2.3 mg/dL, bicarbonate 12 mEq/L, phosphorus 2.3 mg/dL, and anion gap 6 mEq/L. Arterial blood gas showed a pH of 7.35 and pCO2 23 mmHg. Urine glucose was > 500 mg/dL, fractional excretion of phosphate was 56% and fractional excretion of uric acid was 75%. He was diagnosed with Fanconi syndrome due to ICIs (nivolumab and ipilimumab). A kidney biopsy was not done. His renal function returned to baseline after four weeks of corticosteroid treatment. He received intravenous bicarbonate followed by oral bicarbonate for metabolic acidosis.

## Conclusions

AKI is a rare complication of checkpoint inhibitor immunotherapy. The discontinuation of ICIs and treatment with corticosteroids are indicated for patients with severe renal injury but not in all cases. The most common reported pathology is ATIN, but immune complex glomerulonephritis and thrombotic microangiopathy (TMA) have also been observed. ICI-AKI can occur weeks or months after the initiation of treatment with ICIs. Some patients can be cautiously rechallenged with ICIs. A kidney biopsy is required for the diagnosis if an alternative diagnosis, such as acute glomerulonephritis or acute tubular injury, is suspected. Importantly, some patients develop a variety of electrolyte disorders such as hypocalcemia, hyponatremia, hypokalemia, and Fanconi syndrome. These electrolyte disorders can be severe and require regular laboratory monitoring.
